# Diagnostic Infrared Thermography of the Tongue and Taste Perception in Patients with Oral Lichen Planus: Case-Control Study

**DOI:** 10.3390/jcm13020435

**Published:** 2024-01-12

**Authors:** Elena Nicolas-Rodriguez, Eduardo Pons-Fuster, Pia López-Jornet

**Affiliations:** 1 Faculty of Medicine and Odontology, Hospital Morales Meseguer, Clínica Odontológica, Marqués del Los Vélez s/n, 30008 Murcia, Spain; elena.nicolasr@um.es; 2 Departamento de Anatomía Humana y Psicobiología, Faculty of Medicine and Odontology, Biomedical Research Institute (IMIB-Arrixaca), University of Murcia Spain, 30100 Murcia, Spain; eduardo.p.f@um.es

**Keywords:** infrared thermography, oral lichen planus, taste

## Abstract

Oral lichen planus (OLP) is a chronic inflammatory disease of autoimmune origin that affects the skin and mucous membranes. The aim of this study was to assess the effectiveness of infrared thermography (IRT) as a diagnostic tool in patients with oral lichen planus (OLP), as well as disturbances in taste perception, in cases with and without tongue involvement. A case-control study was carried out in a sample of 87 patients divided into three groups: healthy individuals (controls; *n* = 43), OLP patients without involvement of the tongue (*n* = 24), and OLP patients with involvement of the tongue (*n* = 20). The patient symptoms and the clinical characteristics of the lesions were assessed. Four thermal images of the tongue were obtained using IRT: dorsum, right and left lateral surface, and tip of the tongue. General taste perception capacity and subjective sweet, acid, salty, and bitter sensation were evaluated in all three groups. There were no statistically significant differences in the IRT values among the three groups (*p* ≥ 0.05). In the OLP patients with involvement of the tongue, significantly higher values were observed in the mixed forms of the disease (*p* = 0.032). The OLP patients with and without involvement of the tongue showed no significant alterations in taste sensation (*p* = 0.69). IRT may serve as a complementary tool for assessing the activity of OLP with involvement of the tongue. However, more research is needed in this field.

## 1. Introduction

Oral lichen planus (OLP) is a chronic inflammatory disease of autoimmune origin that affects the skin and mucous membranes (mainly oral and genital) and, to a lesser extent, the scalp, nails and other mucous membranes. The disease is characterized by outbreaks, and its underlying etiology remains unclear [1,2]. The prevalence of OLP in the general population is approximately 1.1%, though there are important demographic differences, with a greater frequency of the disease in Europe [3]. The prevalence gradually increases from the fourth decade of life onwards, and women are more often affected than men [4,5]. The oral manifestations of OLP are usually bilateral and symmetrical, though the symptoms and clinical characteristics of the lesions may vary among patients. The lesions typically develop on the cheek mucosa, gums, and tongue, though the palate and lips may also be affected with less frequency. Various clinical types of lesions may be distinguished: reticular, plaque, papular, atrophic, ulcerative/erosive. The most common presentation is reticular OLP, characterized by bilateral keratotic white streaks on the cheek mucosa (called Wickham striae) which are usually asymptomatic [4,5,6,7,8]. On the other hand, the erosive and/or atrophic forms manifest as desquamative, ulcerated and red lesions that tend to cause pain or burning sensation. The symptoms increase with the intake of certain foods, particularly hot, spicy, and acidic foods. Factors that may impact the initiation, perpetuation, or exacerbation of oral lichen planus lesions have been described, including local factors: mechanical (ill-fitting prosthetics, dental edges), chemical (alcohol and tobacco), or biological (bacterial plaque) [6]. The diagnosis is established mainly from the clinical findings, though with histopathological confirmation [5,6,7,8,9,10,11,12,13].

The treatment strategy is conditioned by the patient’s symptoms and the clinical characteristics of the lesions. While reticular OLP is relatively easy to control, erosive OLP is painful and sometimes refractory to treatment, resulting in a negative impact upon patient quality of life [4]. Moreover, the management of erosive OLP is complicated, and no treatment gold standard has been established to date. However, a number of therapeutic approaches have been reported, including the use of systemic corticosteroids, systemic retinoids, calcineurin inhibitors and, recently, drugs targeted against interleukin (IL)-17 and IL-23. The main objective of treatment is to reduce and control the symptoms, eliminate the atrophic/erosive lesions, and lessen the risk of malignization. In this respect, OLP can transform into oral squamous cell carcinoma (OSCC), with an estimated malignization rate of 1.14–1.37% [14].

When used in a clinical setting, thermography is a complementary imaging technique that detects, records and produces an image (thermogram) of the temperature of the surface of the skin and/or the thermal patterns of the patient. Thermography does not use ionizing radiation, and the examination causes no harm to the patient. An abnormal body temperature is a natural indicator of disease. Infrared thermography (IRT) is a rapid, contactless and noninvasive alternative to the use of conventional clinical thermometers for monitoring body temperature. It has been successfully used in the diagnosis of breast cancer, diabetic neuropathy, peripheral vascular disorders, and in odontology [15,16,17,18,19,20,21,22,23,24,25,26,27].

The noninvasive techniques used to date for the diagnosis of OLP are still the subject of research, and include optical fluorescence imaging and optical coherence tomography (OCT). Lichen planus is a chronic disease characterized by exacerbations and remissions. Continuous supervision and follow-up are thus required, and in the event of changes in the lesions, a biopsy is indicated as the gold standard approach [2,11]. The activity and impact of OLP are typically assessed on a clinical basis, using subjective scoring systems that provide useful information but which may benefit from the incorporation of more objective and quantitative measurement techniques such as IRT—thereby affording a more complete and precise evaluation. IRT is a contactless technique that objectively measures temperature changes. It is therefore of interest to carry out studies to assess OLP with involvement of the tongue and the activity of the disease in relation to variations in temperature [22,23].

Tongue temperature is clinically useful, because the tongue has an abundant blood supply, and its surface temperature reflects the internal temperature, transmitted by the blood flow [22]. The temperature of the tongue measured by IRT shows correlations to patient age and gender, and there are also differences in temperature depending on the zone: the temperature is highest at the root of the tongue and gradually decreases towards the tip [22,23,24,25,26,27].

In the context of OLP, thermography may be an interesting tool for study and follow-up of the disease, contributing to better understanding of the disorder and the development of more effective control strategies. The working hypothesis of the present study was that the thermography findings and taste sensation may be altered in OLP patients with involvement of the tongue versus OLP patients without involvement of the tongue (positive controls) and healthy individuals (negative controls). Specifically, the present study was carried out to analyze the use of IRT and taste perception in OLP patients with and without involvement of the tongue.

## 2. Materials and Methods

The study protocol was approved by the Research Ethics Committee of the University of Murcia (Murcia, Spain) (ID: 4208/2023), and was carried out following the STROBE (Strengthening the Reporting of Observational Studies in Epidemiology) statement. The study was conducted in compliance with the Declaration of Helsinki on research in human subjects.

### 2.1. Participants

Patients from the Department of Oral Medicine of the Dental Clinic of Morales Meseguer Hospital (University of Murcia) were consecutively included in the study. Informed consent was obtained from all the participants, and personal data protection was observed.

Patients with OLP were diagnosed based on the clinical findings, with histopathological confirmation, in accordance with the consensus document on oral potentially malignant disorders [11]. The control group consisted of healthy individuals without oral lesions, and with sociodemographic characteristics similar to those of the OLP patients.

The exclusion criteria in all groups were: decompensated systemic disease, a history of oral cancer, infection and/or trauma of the neck, face or oral cavity, the use of vasodilator drugs, pregnant women, and individuals under 18 years of age.

### 2.2. Data Collection

**Figure 1:**
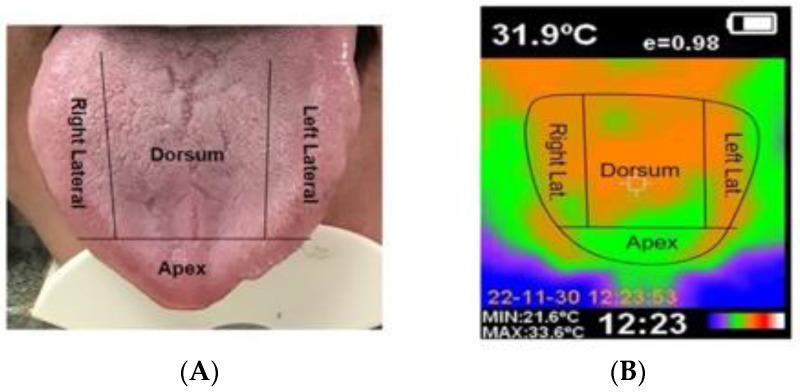
(**A**) Dorsal Lingual; (**B**) Thermograpgic Image.

**Figure 2:**
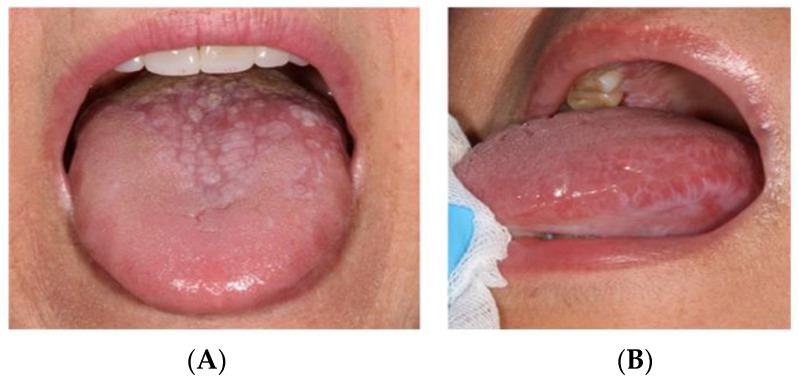
(**A**) Mixed form of oral lichen planus; (**B**) Reticular form of oral lichen planus.

Data collection was carried out between November 2022 and June 2023. An ad hoc questionnaire was used to record demographic data (age and gender). The oral examination recorded the clinical characteristics of OLP, and a subjective evaluation of the patient’s taste alterations and symptoms was made. IRT was used for the thermal measurement of the different zones of the tongue, with the recording of thermal and digital images (Figure 1, we can see the division of the tongue dorsum: right side of the tongue; left side of the tongue; tip of the tongue; and center of the tongue, followed by the thermographic image). In Figure 2, we can observe various clinical presentations of oral lichen planus on the tongue.

The lesions were clinically classified according to their phenotype as follows: hyperkeratotic, atrophic-erosive, or mixed. Hyperkeratotic lesions comprised reticular, papular and/or plaque forms, representing the so-called “white lesions”, while atrophic-erosive lesions comprised erosive, atrophic, and ampullar clinical lesions, representing the so-called “red lesions” [6].

For evaluation purposes, the tongue was divided into four zones: dorsum, right and left lateral surface, and tip of the tongue. Each zone in turn was phenotypically classified as presenting no lesions, hyperkeratotic lesions, atrophic-erosive lesions, or mixed lesions.

### 2.3. Subjective Evaluation of Symptoms

Patient symptoms assessment was carried out with a questionnaire, scoring burning sensation and dry mouth with a visual analogue scale (VAS) from 0–10 (0 = none, 10 = maximum). A qualitative evaluation was also made of taste alteration, where the patients answered the question: “Does food taste different?”, using the options: “never”, “rarely”, “sometimes”, “often”, or “always”. This was done both in general terms and with reference to each individual taste sensation (sweet, acid, salty, and bitter) [28].

### 2.4. Recording of Thermographic Images

Infrared thermography was carried out as described elsewhere [20], in a room with controlled temperature (21 °C) and relative humidity (50–60%). The patients were allowed to rest for 10–15 min to adjust to the conditions of the dental clinic, and IRT was performed at least one hour after the last hot or cold food and/or drink intake.

With the patient in a sitting position, the examiner maintained a distance of 15–20 cm between the target body surface (tongue) and the thermographic device. The temperatures of each zone were recorded in the absence of any physical contact with the patient, in order to avoid heat transfer between bodies and distortion of the measurements. Three thermal values were recorded in each tongue zone (dorsum, right and left lateral surface, and tip of the tongue) and four thermal images were obtained (one for each zone), together with a digital image [24].

The tongue temperature recordings were made using a manual thermographic camera (model HT–02D, Hti Dongguan Xintai Instrument Co., Ltd., Dongguan, China), with a measurement precision of ± 2% and a thermal sensitivity of 0.3 °C. The operating wavelength was between 8–11.5 µm, with a field of view of 33° × 33°, and a minimum focal distance of 0.5 m. Emissivity was adjustable between 0.1–1.0, and the image resolution was 32 × 32 (1024 pixels).

Emissivity is the ratio between the radiation emitted by the surface of a body and the radiation emitted by a black body at the same temperature. In the case of the human body, emissivity is considered to range between 0.94–0.99. In the present study the value was established as 0.95. A rainbow color palette was used to obtain the images, representing a color scale from blue to red.

Considering a sample size of 24 individuals per group and an effect size of 0.5, the statistical power of the study was estimated to be about 80%. This suggests a reasonable probability of detecting the specific temperature difference we consider to be clinically significant, based on the design of our study.

### 2.5. Statistical Analysis

A descriptive analysis was made, with calculation of the mean and standard deviation (SD) for quantitative variables, and frequencies and percentages for qualitative variables. As the sample size was under 50 patients, the relationship between variables was explored using Spearman’s correlation coefficient. Group comparisons were based on contingency tables for qualitative variables, while in the case of quantitative variables the Mann-Whitney U-test was used for the comparison of two groups, and analysis of variance (ANOVAS) for more than two groups. Differences between groups with the ANOVA test were contrasted using the Tukey post hoc test. The effect size was estimated according to Domínguez-Lara [29] for each statistic. Statistical significance was considered as *p* ≤ 0.05. The SPSS version 28.0 statistical package was used throughout.

## 3. Results

### Study Sample

The study sample consisted of 87 subjects divided into three groups: healthy individuals (control group; *n* = 43, 49.4% of the sample), OLP patients without involvement of the tongue (*n* = 24, 27.3% of the sample), and OLP patients with involvement of the tongue (*n* = 20, 23% of the sample). The sociodemographic data and disease characteristics of the subjects diagnosed with OLP are shown in Table 1. The mean age of the participants was 60.43 ± 10.7 years, with a predominance of females in all three groups, representing 75.9% of the total sample (*n* = 66), while males accounted for 34.1% (*n* = 21).

The comparison of the three groups regarding temperature measured in each zone of the tongue is shown in Table 2. There were no statistically significant differences in the IRT values among the groups. The temperature was found to be highest on the dorsum of the tongue, followed by the lateral sides of the tongue and the tip, in all three groups.

With regard to the symptoms, the OLP patients without involvement of the tongue reported a burning sensation score of 2.17 ± 2.7, while those with involvement of the tongue yielded a score of 2.10 ± 3.4 (*p* = 0.691). In turn, the score referring to dry mouth sensation was 3.17 ± 4.0 and 4.95 ± 4.0, respectively (*p* = 0.126).

In the group corresponding to OLP with involvement of the tongue, on considering the location and frequency of each clinical presentation (Table 3), significant temperature differences were observed (*p* = 0.032) according to the clinical type of disease, with higher values being recorded in the mixed presentations (Table 4).

With regard to taste sensation, the OLP patients with and without involvement of the tongue globally showed no alterations in relation to the four individual taste sensations assessed in the questionnaire. There were no statistically significant differences between the two groups (Table 5).

The correlation analysis between taste alteration and tongue lesions in the OLP patients with involvement of the tongue (Table 6) showed a medium level correlation (r = 0.3–0.7) between the lesions on the left and right side of the tongue. A high and very high correlation was found (r > 0.875) between taste alteration overall and the four individual taste sensations. The results showed no relationship between taste alteration (dysgeusia) and the presence of lesions in any of the different zones of the tongue.

## 4. Discussion

In this study, markedly higher temperature values were recorded in the mixed forms of OLP with involvement of the tongue. However, there were no significant differences in taste sensation among the different groups.

Many studies [17,23,30,31,32,33] have shown that thermal images of the body surface reflect the variability of blood flow and metabolism in a region of interest, and can be used to identify a broad range of disease conditions. Although IRT is not a specific technique and sometimes depends on the patient history and environment, it has become widely accepted for a number of reasons. On the one hand, IRT is a rapid, noninvasive and contactless technique. Thermogram interpretation based on color codes moreover simplifies the study. On the other hand, IRT only records the natural radiation from the body surface, and involves no exposure to ionizing radiation. The technique is therefore adequate for prolonged and repeated use. Lastly, IRT is a real-time technique that allows the monitoring of dynamic temperature variations, and avoids inter-observer variability and subjective bias [23,24,25,26,27].

The systematic review carried out by Almeida et al. [32] underscores the versatility and potential of IRT as a tool for the diagnosis and evaluation of many disorders affecting the muscles of the head and neck. It is useful for diagnosing clinical conditions associated with pain phenomena, and could play a role in defining clinical treatment and follow-up strategies. Furthermore, IRT is potentially able to predict changes in the musculoskeletal system of the head and neck.

Other authors [31] have reported significant temperature differences when evaluating facial cellulitis and dental abscesses. There are temperature differences between the affected and the non-affected side, and such differences are moreover greater in cases of cellulitis than in patients with dental abscesses.

Thermography has also been successfully used to diagnose periapical inflammatory lesions [32]. The data obtained show the highest temperature values to correspond to acute apical lesions. On the other hand, thermography could detect inflammatory reactions during the preclinical phase—thus facilitating an early diagnosis.

In a recent study, Wziatek-Kuczmik et al. [33] used thermography as a diagnostic technique to identify oral infectious foci in patients with systemic diseases. The authors recorded statistically significant differences between groups, and concluded that the technique is of potential use in the prevention and diagnosis of systemic disorders.

In our study, the temperature values were higher on the dorsum of the tongue than on the lateral zones, whereas similar values were found for both sides (i.e., left and right). The lowest values in turn corresponded to the tip of the tongue. This can be explained by the magnitude of the blood supply in each zone of the tongue mucosa, and the relationship between blood flow and local temperature [22,23].

Bermejo-Fenoll et al. [33] carried out a morphometric study of the tongue in patients with OLP. Of a total of 236 patients, 111 presented lesions on the dorsum, and the results showed that in 73% of cases the percentage affected area was less than 50%—with no significant correlation being observed between the affected tongue surface and the age of the patients.

Subjective taste sensation (both overall and according to each of the individual taste sensations) showed no differences between OLP patients with and without involvement of the tongue. Suter et al. [28] reported lower overall taste sensation in OLP patients with involvement of the tongue. This decrease could be due to the lower density of taste papillae on the dorsum of the tongue affected by OLP lesions, with atrophy, erosion, ulceration or hyperkeratosis. According to Suter [34], the type of OLP lesion does not appear to be the determining factor in altered taste function among patients with OLP.

In general, reduced taste sensation in elderly subjects is attributed to a lower number of taste papillae [35]. In our study, the patients in both groups with OLP were of similar age; thus, age was unlikely to have been a confounding factor. The study population came from the same ethno-cultural background, and the gender distribution was equal between groups. It is important to note that the measurements were obtained before the patients received active treatment, since taste alterations are usually reported in the course of the treatment of symptomatic OLP, due to the side effects of the prescribed drugs [36].

Lichen planus is a chronic disease that requires continuous monitoring for adequate control [37,38], and in this regard temperature changes of the tongue could be related to the activity of the disease. The data obtained could help to better understand the disorder and develop more effective control strategies. In the context of OLP, oral thermography could constitute an interesting noninvasive monitoring tool, and also contribute towards more personalized care based on scientific evidence, in patients diagnosed with this disease.

In summary, while infrared thermography shows promise as a monitoring tool for OLP, its ability to directly detect genetic variations may be limited. Instead, it can provide valuable information about the inflammatory and physiological changes associated with the disease.

Our study has some limitations. In effect, its observational design means that interventional follow-up studies are needed to determine whether the model based on IRT results in a change in the outcome of the lesions. On the other hand, taste alteration was assessed on a subjective basis, without using objective quantitative tests such as taste strips. Given the preliminary nature of the investigation, a larger sample size would be advisable. On the other hand, it must be mentioned that effective application of IRT and interpretation of the thermograms requires adequate operator training [17]. Further studies are needed to simplify and standardize the technique, in order to improve interpretability in identifying the disease.

## 5. Conclusions

In sum, IRT may offer valuable information, as it is an objective and noninvasive monitoring tool allowing rapid data acquisition that can be used along with other evaluation techniques, and may serve as a complement for assessment of the activity of OLP with involvement of the tongue. Further research is needed to fully understand the potential and limitations of this diagnostic approach.

## Figures and Tables

**Table 1:** General characteristics of the sample.

Variables		Total		Control		OLP		OLP Tongue		*p* Value
		*n*	%	*n* 43	49.4%	*n* 24	27.3%	*n* 20	23%	
Sex	Female	66	75.9	34	79.1	15	62.5	17	85.0	0.164
	Male	21	24.1	9	20.9	9	37.5	3	15.0	
		Mean	SD	Mean	SD	Mean	SD	Mean	SD	
Age		60.43	10.7	57.02	10.5	63.58	6.4	63.95	13.3	0.012 *
OLP Evolution	<6 months	11	25.0	-	-	1	4.2	10	50.0	0.001 ***
	6 months–2 years	11	25.0	-	-	6	25.0	5	25.0	
	3–5 Years	7	15.9	-	-	7	29.2	0	0	
	6–10 Years	6	13.6	-	-	6	25.0	0	0	
	>10 Years	9	20.5	-	-	4	16.6	5	25.0	
OLP Clinical form	Reticular/	14	31.8	-	-	9	37.5	5	25.0	0.111
	Atrophic/Erosive	11	25.0	-	-	8	33.3	3	15.0	
	Mixes	19	43.2	-	-	7	29.2	12	60.0	

Note: *n*—sample size; %—frequency; SD—standard deviation; * *p* < 0.05; *** *p* < 0.001.

**Table 2:** Distribution of lingual temperature according to study group.

Variables	Control		OLP		OLP Lingual		F(gl)	*p* Value	η^2^
	Mean	SD	Mean	SD	Mean	SD			
Tª M Dorsum	33.27	1.0	32.85	0.9	33.28	0.7	1.97(2)	0.146	0.045
Tª M Lateral right	32.79	1.0	32.54	0.7	32.71	0.7	0.67(2)	0.515	0.016
Tª M Lateral Left	32.61	1.0	32.12	0.8	32.17	0.7	2.97(2)	0.057	0.066
Tª M Apex	31.37	1.2	31.01	0.8	31.43	0.8	1.18(2)	0.313	0.027

M—mean; SD—standard deviation.

**Table 3:** Distribution and clinical form of lingual lesions in patients with lingual OLP (*n* = 20).

Variables		*n*	%
Dorsum	No lesion	11	55.0
	Reticular/plaque	3	15.0
	Atrophic/Erosive	3	15.0
	Mixed	3	15.0
Lateral right	No lesion	5	25.0
	Reticular/plaque	5	25.0
	Atrophic/Erosive	5	25.0
	Mixed	5	25.0
Lateral left	No lesion	6	30.0
	Reticular/Plaque	5	25.0
	Atrophic/Erosive	5	25.0
	Mixed	4	20.0
Tip	No lesion	18	90.0
	Reticular/Plaque	1	5.0
	Atrophic/Erosive	1	5.0
	Mixed	-	-

Note: *n*—sample size; %—frequency.

**Table 4:** Comparative study of lingual temperatures among different clinical forms of presentation of oral lichen planus and control.

Variables	No Lesion (*n* = 40)	Reticular/ Plaque (*n* = 14)	Atrophic/Erosive (*n* = 14)	Mixed (*n* = 12)	Sig.	η^2^
Temperature	32.12 (1.0)	32.51 (0.9)	32.50 (1.1)	33.07 (1.0)	0.032	0.109

Note: η^2^—Eta squared; 0.04—minimum necessary effect; 0.25—moderate effect; 0.64—strong effect.

**Table 5:** Taste alterations in patients with OLP.

Variable		OLP		OLP Lingual		χ^2^(gl)	*p* Value	V
		*n*	%	*n*	%			
Taste alteration	Never	16	66.7%	13	65.0%	1.83(3)	0.609	0.204
	Sometimes	3	12.5%	2	10.0%			
	From time to time	4	16.7%	2	10.0%			
	Often	1	4.2%	3	15.0%			
Sweet	Never	18	75.0%	15	75.0%	0.24(3)	0.97	0.075
	Sometimes	2	8.3%	1	5.0%			
	From time to time	3	12.5%	3	15.0%			
	Often	1	4.2%	1	5.0%			
Salty	Never	18	75.0%	14	70.0%	0.68(3)	0.879	0.124
	Sometimes	1	4.2%	2	10.0%			
	From time to time	3	12.5%	2	10.0%			
	Often	2	8.3%	2	10.0%			
Sour	Never	18	75.0%	14	70.0%	0.62(3)	0.892	0.118
	Sometimes	2	8.3%	1	5.0%			
	From time to time	3	12.5%	4	20.0%			
	Often	1	4.2%	1	5.0%			
Bitter	Never	17	70.8%	15	75.0%	2.93(3)	0.403	0.258
	Sometimes	3	12.5%	0	0.0%			
	From time to time	3	12.5%	4	20.0%			
	Often	1	4.2%	1	5.0%			

Note: V—Cramer’s V; 0.10—small effect; 0.30—medium effect; 0.50—large effect.

**Table 6:** Correlation between taste alterations and lingual lesions in patients with lingual OLP (*n* = 20).

Variables	1	2	3	4	5	6	7	8	9
1. Taste alteration	--								
2. Sweet	0.875 **	--							
3. Salty	0.927 **	0.925 **	--						
4. Sour	0.936 **	0.934 **	0.990 **	--					
5. Bitter	0.884 **	0.996 **	0.919 **	0.937 **	--				
6. Dorsum	0.140	0.133	0.086	0.046	0.119	--			
7. Lateral right	−0.146	0.010	−0.077	−0.120	−0.010	0.230	--		
8. Lateral left	0.040	0.287	0.192	0.149	0.256	0.080	0.661 **	--	
9. Apex	0.149	0.099	0.057	0.125	0.154	−0.286	0.022	−0.089	--

Note: r < 0.3 low ratio; r between 0.3–0.7 average; r between 0.7–0.9 high; r > 0.9 very high ** *p* < 0.01.

## Data Availability

The datasets generated during and/or analyzed during the current study are available from the corresponding author on reasonable request.
